# The sternocleidomastoid muscle flap for the prevention of Frey syndrome and cosmetic deformity following parotidectomy: A systematic review and meta-analysis

**DOI:** 10.3892/ol.2013.1179

**Published:** 2013-02-05

**Authors:** DONG YAN LIU, XIAO JIAO TIAN, CHENG LI, SHAO SHAN SUN, YING HUI XIONG, XIAN-TAO ZENG

**Affiliations:** 1 Departments of Stomatology, Taihe Hospital, Hubei University of Medicine, Shiyan 442000;; 2 Neurosurgery, Taihe Hospital, Hubei University of Medicine, Shiyan 442000;; 3 Department of Oral and Maxillofacial Surgery, School and Hospital of Stomatology, Wuhan University, Wuhan 430079;; 4 Department of Stomatology, People’s Hospital of Beijing Daxing District, Capital Medical University, Beijing 102600, P.R. China

**Keywords:** sternocleidomastoid muscle flap, Frey syndrome, gustatory sweating syndrome, cosmetic deformity, parotidectomy, systematic review

## Abstract

Approximately 34–86% of neoplasms of the salivary glands are located in the parotid gland and parotidectomy is the first-line treatment for parotid gland tumors. Frey syndrome and cosmetic deformity are common complications experienced by patients following parotidectomy and the sternocleidomastoid muscle flap (SCMF) is used to prevent them. Numerous studies have been performed to examine the effectiveness of the SCMF for the prevention of cosmetic deformity and Frey syndrome, however, they provide contradictory results and possess small sample sizes with consequently low statistical power. In order to evaluate the effectiveness of the SCMF for the prevention of Frey syndrome and cosmetic deformity following parotidectomy, we performed a systematic review and meta-analysis based on published randomized controlled trials (RCTs), which were identified using PubMed and CNKI databases, and references of studies up to August 2012 were included. Using these criteria, we yielded 11 RCTs. Following an independent assessment of the methodological quality of these studies and the extraction of data, a systematic review and meta-analysis was conducted. The results of the meta-analysis demonstrated that there was a significant trend towards a lower risk of objective incidence [67%; risk ratio (RR), 0.33; 95% confidence interval (CI), 0.16–0.67; P<0.01] and subjective incidence (66%; RR, 0.34; 95% CI, 0.16–0.75; P= 0.01) of Frey syndrome in the SCMF group. The sensitivity analysis also indicated that this result was significant. Due to the considerable variation between the included studies, a meta-analysis was not applicable to assess cosmetic deformity. Two RCTs demonstrated that the difference between the SCMF and no SCMF group was not statistically significant, while the other seven RCTs detected a statistically significant difference between the two groups. Publication bias was detected. In conclusion, based on currently available evidence, the use of the SCMF is benefical for the prevention of Frey syndrome, however, whether it is also benefical for cosmetic deformity remains inconclusive.

## Introduction

Approximately 34–86% of neoplasms of the salivary glands are located in the parotid gland (1,2). Parotidectomy is the first-line treatment for parotid gland tumors (3,4). However, complications experienced following surgery significantly reduce patients’ quality of life (5). The most common complications are cosmetic deformity and Frey syndrome.

Owing to all or part of the parotid gland being removed, facial depression is observed in almost 100% of patients who have undergone parotidectomy. Frey syndrome was first described by Łucja Frey in 1923 (6), with an incidence of between 11 and 95% (7,8). It is characterized by flushing or sweating on one side of the forehead, face, scalp and neck occurring soon following the ingestion of food, in response to salivatory stimulation (9).

The use of autogenous tissue interposition for the prevention of Frey syndrome and cosmetic deformity during parotidectomy are considered as simple, safe and effective approaches by clinicians. The sternocleidomastoid myocutaneous flap (SCMF) is one of the most commonly used autogenous tissues (10). Numerous studies have been performed to examine the effectiveness of the SCMF for the prevention of cosmetic deformity and Frey syndrome, however, they provide contradictory results and have small samples sizes with consequently low statistical power.

In 2010, Sanabria *et al* (10) conducted a meta-analysis investigating the effectiveness of the SCMF for the prevention of cosmetic deformity and Frey syndrome. The study included two randomized controlled trials (RCTs) (11,12). At the time of writing this manuscript, eleven RCTs have been published. A comprehensive systematic review and meta-analysis is therefore required to provide an updated review of the effectiveness of the SCMF for the prevention of cosmetic deformity and Frey syndrome.

## Materials and methods

### Report terms

We attempted to follow the proposed PRISMA (Preferred Reporting Items for Systematic Reviews and Meta-Analyses) guidelines (13) to report the present systematic review and meta-analysis.

### Literature search

We conducted a PubMed and CNKI database search in August 2012 for relevant studies that examined the effectiveness of the SCMF for the prevention of cosmetic deformity and/or Frey syndrome. The following search terms were used: i) Frey’s syndrome, Frey syndrome, gustatory sweating, auriculotemporal syndrome, cosmetic deformity, facial depression and cosmetic disfigurement; ii) sternocleidomastoid. These two search terms were combined using the Boolean operator ‘and’. No restrictions were imposed. In addition, we examined the reference lists of the retrieved RCTs and recently published reviews.

### Study selection

We conducted an initial screening of titles or abstracts. Following this, we performed a second screening based on full-text review. Studies were considered eligible if they met the following criteria: i) the study design was an RCT; ii) the study included patients with benign or malignant parotid tumors who underwent partial or total parotidectomy with facial nerve preservation, and without a history of previous surgical procedures in the parotid area or previous radiotherapy; iii) interventions included the SCMF, and the control group constituted no SCMF or other prophylactic measures; iv) the main outcome was incidence of cosmetic deformity and/or Frey syndrome, determined with subjective or objective measures; v) data of each outcome were reported or obtained by contacting the corresponding author of the study.

### Data extraction

Two authors (XJ Tian and YH Xiong) independently extracted the following data for each eligible study: first author’s last name, year of publication, site of origin, sample size, characteristics of the SCMF and control groups, outcome evaluation methods, length of follow-up, incidence of cosmetic deformity and Frey syndrome. Any disagreements were resolved by consulting a third author (XT Zeng).

### Assessment of methodological quality

The methodological quality of each study was assessed using the Cochrane collaboration’s tool for assessing risk of bias (14), which contains the following seven criteria: i) details of the randomization method; ii) allocation concealment; iii) blinding of participants and personnel; iv) blinding of outcome assessment; v) incomplete outcome data; vi) selective outcome reporting and vii) other sources of bias. Each study was assessed by two authors (DY Liu and XJ Tian) independently and any disagreements were resolved by consulting a third author (XT Zeng).

### Data synthesis and analysis

We calculated risk ratios (RRs) and 95% confidence intervals (CIs) for all studies with sufficient data. Heterogeneity was examined using the Cochrane Q test and quantified with the I^2^ statistic (15). The value of the I^2^ statistic was used to select the appropriate pooling method: if the I^2^ value was <50%, the fixed-effects meta-analysis was applied; if the I^2^ value was ≥50%, the random-effects meta-analysis was used.

In the presence of heterogeneity, we performed sensitivity analyses by removal of each study in turn in order to examine the robustness of the main results. Potential publication bias was investigated by visual assessment using a funnel plot and further examined using a combination of the Egger regression test (16) and the ‘trim and fill’ method (17).

Statistical analyses were conducted with Comprehensive Meta-Analysis software, version 2.2 (Biostat, Englewood, New Jersey, USA) (18). For all comparisons, except those for heterogeneity, P<0.05 was considered to indicate a statistically significant result. All tests were two-sided.

## Results

### Search results

**Figure 1:**
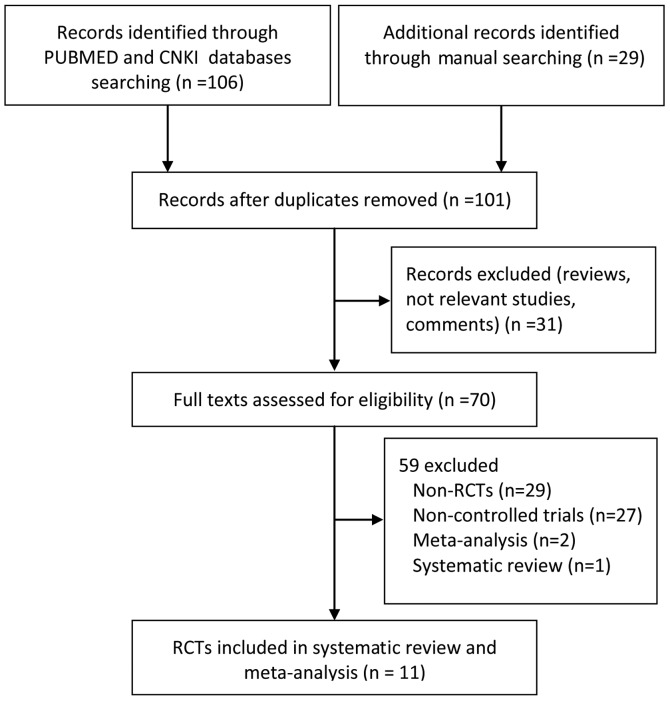
Summary of the study selection process. RCTs, randomized controlled trials.

An initial search yielded 135 potentially relevant studies and 11 RCTs (11,12,19–27) were selected for the purpose of our analysis. Fig. 1 depicts a flowchart showing the study selection process and their characteristics are listed in Table I.

### Methodological quality

Table II shows the quality of RCTs according to the Cochrane collaboration’s tool. It refers to randomization only, lacking information with regard to allocation concealment and blinding; however, no incomplete outcome data, no selective outcome reporting and other sources of bias were observed. Therefore, there was a moderate risk of bias.

### Frey syndrome

**Figure 2:**
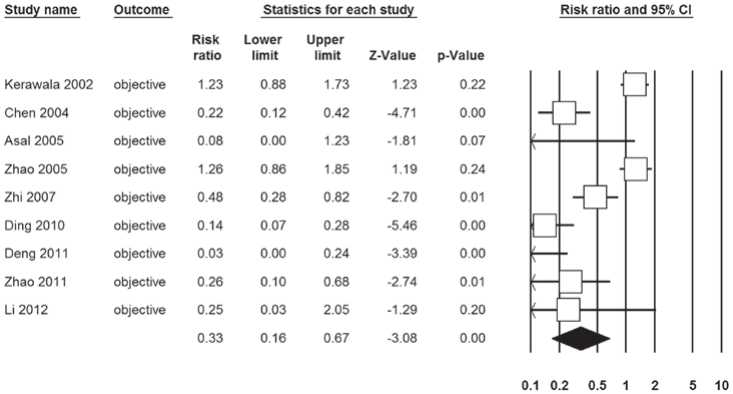
Forest plot of the risk ratios and 95% CI of the incidence of objective Frey syndrome. CI, confidence interval.

**Figure 3:**
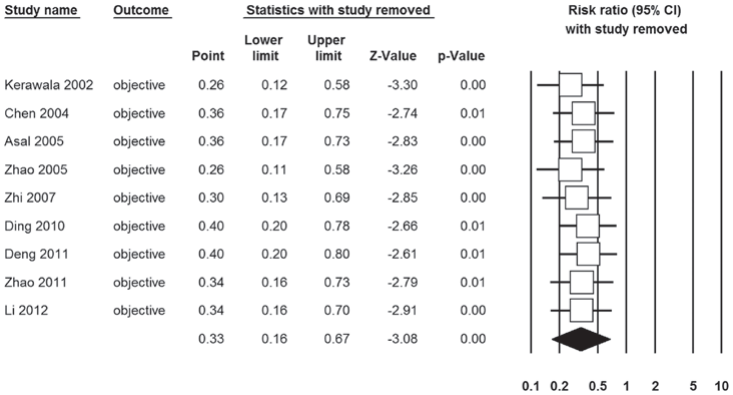
Forest plot of the risk ratios and 95% CI of the incidence of objective Frey syndrome following sensitivity analysis performed by removing each study consecutively. CI, confidence interval.

Nine RCTs (11,12,19–22,24,26,27) reported the incidence of objective Frey syndrome by performing the starch-iodine test. A significant heterogeneity was observed (I^2^=88.79%, P<0.10), therefore we used a random-effects model. The meta-analysis demonstrated that the SCMF markedly decreased the risk of incidence of Frey syndrome (67%; RR, 0.33; 95% CI, 0.16–0.67; P<0.01; Fig. 2). Sensitivity analysis was performed by sequential removal of each study and the significance of pooled RR was not influenced by omitting any single study, suggesting that the result of this meta-analysis was stable (Fig. 3).

**Figure 4:**
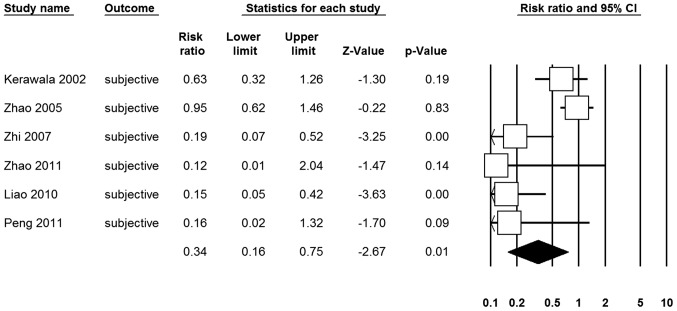
Forest plot of the risk ratios and 95% CI of the incidence of subjective Frey syndrome. CI, confidence interval.

**Figure 5:**
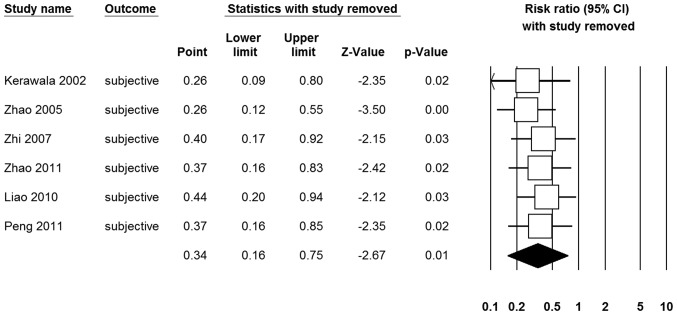
Forest plot of the risk ratios and 95% CI of the incidence of subjective Frey syndrome following sensitivity analysis performed by removing each study consecutively. CI, confidence interval.

Seven RCTs (11,12,19,21,23,26,28) reported the subjective incidence of Frey syndrome. A significant heterogeneity (I^2^=74.24%, P<0.10) was observed, therefore, the random-effects model was used. The result also demonstrated that there was a significant correlation towards a lower risk of incidence in the SCMF group (66%; RR, 0.34; 95% CI, 0.16–0.75; P= 0.01; Fig. 4). The sensitivity analysis also indicated this result was significant (Fig. 5).

### Cosmetic results

Nine RCTs (11,12,19–24,27) observed cosmetic deformity. Due to the considerable variation among included studies, meta-analysis was not applicable. Two RCTs (11,12) demonstrated that the differences between the SCMF and no SCMF group were not statistically significant, while the other seven RCTs demonstrated that the cosmetic appearance was statistically significant for the SCMF group compared with the no SCMF group. Table III indicates a qualitative analysis of the evidence.

### Publication bias

**Figure 6:**
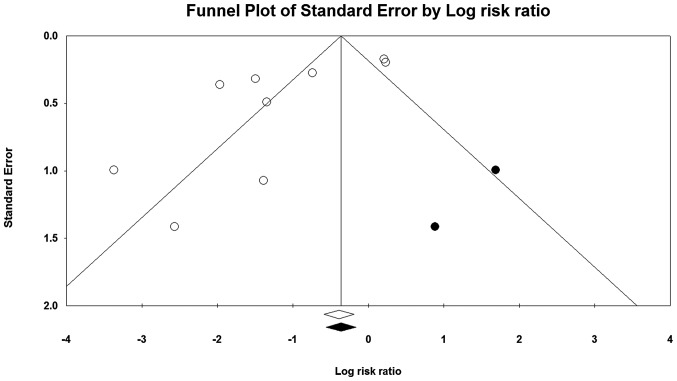
Filled funnel plot with pseudo-95% CIs for the evaluation of objective Frey syndrome. A circle represents a study, while a black spot represents an unpublished study that is required to negate the results of the meta-analysis. CI, confidence interval.

Fig. 6 demonstrates that the funnel plot was asymmetrical (based on the evaluation of objective Frey syndrome), which indicated that publication bias existed (white circles). The Egger linear regression also detected moderate publication bias among studies (Egger, P=0.023). As evidence of bias may be due to inadequate statistical power, we used a non-parametric method of ‘trim and fill’ to estimate two possible missing studies (black spots in Fig. 6), the estimated RR including the ‘missing’ studies was not substantially different from our estimate with an adjustment for the missing studies (RR, 0.43; 95% CI, 0.22–0.88).

## Discussion

In 1927, Andre Thomas described the pathophysiology of Frey syndrome as the aberrant regeneration of sectioned para-sympathetic fibers, which regrow to innervate the vessels and sweat glands of the skin overlying the parotid (29). Accepting this pathophysiology and mechanism, oral and maxillofacial surgeons proposed to interpose any tissue between the parotid bed and the skin, including the temporoparietal fascia rotational flap (30), the superficial muscular aponeurotic system (SMAS) (31), the SCMF and AlloDerm (32), with the aim of inhibiting aberrant innervation in order to prevent Frey syndrome.

Compared with other autogenous tissue flaps, the SCMF has several advantages: i) it is easy to rotate into the parotid region without another incision; ii) it is long enough to cover all the branches of the facial nerve; iii) it decreases the depression of the surgical area following parotid gland resection; iv) there is a low risk of necrosis of the flap due to its vascularization and v) there is a low risk of complications, primarily, spinal accessory nerve injury (10). Compared with AlloDerm, the major advantage of the SCMF is that it is more cost-effective.

The first RCT of the SCMF was published by Kerawala *et al* (11) in 2002. Following that, a number of additional RCTs have been published. However, the results are inconsistent. In 2010, Sanabria *et al* (10) performed a meta-analysis of the SCMF for the prevention of Frey syndrome, including two RCTs and nine non-RCTs. The authors concluded that the result of their meta-analysis was inconclusive with regard to the use of the SCMF as an intervention for the prevention of Frey syndrome following parotid surgery. It is widely considered that a non-RCT design introduces a higher degree of bias compared with a RCT design. Therefore, we conducted a meta-analysis which only included RCTs in order to obtain a more accurate result. The meta-analysis demonstrated that the SCMF is capable of clearly decreasing the incidence of objective and subjective Frey syndrome. The sensitivity analysis indicated that the result was robust.

Some studies have indicated that the SCMF evidently improves cosmetic appearance compared with no flap and some studies have indicated that this function was limited. Due to the considerable variation of the assessment methods used among the included studies, a meta-analysis was not performed. Our systematic review did not obtain a conclusive result.

However, there are some limitations with regard to our systematic review and meta-analysis that should be stated. Firstly, the sample size of the studies contributing a significant amount of data to this meta-analysis was small, therefore, we were not able to adequately assess the effects of prevention. Secondly, the methodological quality of the included RCTs demonstrated a moderate risk of bias, and a lack of information with regard to randomization, allocation concealment and blinding (some RCTs) may have introduced bias. Thirdly, a significant heterogeneity between RCTs existed, although sensitivity analysis revealed that it did not exert a significant influence. However, its potential influence should be considered. Fourthly, publication bias was detected, the ‘file-drawer’ effect may exist and our ‘trim and fill’ analysis also estimated two possible missing studies, which indicated that studies that reported negative results may be more likely to remain unpublished. Lastly, a meta-analysis of the cosmetic result was not conducted, so we are not able to obtain a quantitative result for this outcome.

For future studies, we suggest that the same measurment tools are applied, including the 10-cm visual analog scale (11) and blinding to the evalution of the cosmetic result. We also suggest to perform RCTs to compare the effectiveness of the SCMF and AlloDerm, as AlloDerm is presently widely used for the prevention of Frey syndrome (32). We recommend the use of the starch-iodine test and blinding in studies with regard to Frey syndrome in future studies. As the method of parotidectomy (4) and prognosis (33) are influenced by benign and malignant tumors, diagnosis of parotid gland lesions prior to parotidectomy is important. A well accepted, safe, reliable, minimally invasive and cost-effective method, including fine needle aspiration cytology (34) is recommended.

In conclusion, based on currently available evidence, the use of the SCMF is beneficial for the prevention of Frey syndrome, however, whether it is additionally benefical for cosmetic deformity remains inconclusive.

## Figures and Tables

**Table I:** Characteristics of included RCTs.

Author (ref.)	Year	Country	Sample size (T/C)	Age (years)	Gender (male:female)	Intervention		Follow-up	Outcome
						T	C		
Kerawala *et al* (11)	2002	UK	21/15	range, 26–81	23:13	SCMF	Blank	1–6 years	Cosmetic result; Frey syndrome (objective and subjective)
Chen and Yang (20)	2004	China	108/64	range, 14–78 mean, 43.2	96:76	SCMF	Blank	6 months–5 years	Cosmetic result; Frey syndrome (objective)
Asal *et al* (12)	2005	Turkey	12/12	mean, 49 (T) mean, 50 (C)	7:5/5:7	SCMF	Blank	9–48 months	Cosmetic result; Frey syndrome (objective and subjective)
Zhao *et al* (19)	2005	China	57/33	range, 12–79 mean, 33.92	32:58	SCMF	Blank	2 years	Cosmetic result; Frey syndrome (objective and subjective)
Zhi *et al* (21)	2007	China	38/47	range, 14–68 mean, 35.2	32:53	SCMF	Blank	1 year	Cosmetic result; Frey syndrome (objective and subjective)
Ding *et al* (22)	2010	China	60/60	range, 20–83 mean, 53.6	69:51	SCMF	Blank	18 months–5 years	Cosmetic result; Frey syndrome (objective)
Liao *et al* (23)	2010	China	48/20	mean, 45 (T) mean, 48 (C)	26:22/11:9	SCMF	Blank	6 months–3 years	Cosmetic result; Frey syndrome (subjective)
Deng *et al* (24)	2011	China	52/95	range, 21–72 mean, 52.5	NA	SCMF	Blank	6 months–5 years	Cosmetic result; Frey syndrome (objective)
Peng and Chen (25)	2011	China	22/23	range, 19–72	26:19	SCMF	Blank	3 months–2 years	Frey syndrome (subjective)
Zhao *et al* (26)	2011	China	23/15	range, 18–65	NA	SCMF	Blank	1 year–25 months	Frey syndrome (objective and subjective)
Li and Xu (27)	2012	China	20/20	mean ± SD, 35.3±4.6	22:18	SCMF	Blank	10 months–3 years	Cosmetic result; Frey syndrome (objective)

NA, not avaliable; SCMF, sternocleidomastoid myocutaneous flap; Blank, no flap and direct suture; RCTs, randomized controlled trials; T, sternocleidomastoid myocutaneous flap groups; C, control groups; SD, standard deviation.

**Table II:** Quality of included RCTs according to the Cochrane collaboration’s tool.

Author (ref.)	Year	Randomization method	Allocation concealment	Incomplete outcome data	Blinding of participants and personnel	Blinding of outcome assessment	Selective outcome reporting	Other sources of bias
Kerawala *et al* (11)	2002	Low	Unclear	Low	Low	Low	Low	Low
Chen and Yang (20)	2004	Unclear	Unclear	Unclear	Low	Low	Low	Low
Asal *et al* (12)	2005	Unclear	Unclear	Low	Low	Low	Low	Low
Zhao *et al* (19)	2005	Unclear	Unclear	Unclear	Unclear	Low	Low	Low
Zhi *et al* (21)	2007	Unclear	Unclear	Unclear	Low	Low	Low	Low
Ding *et al* (22)	2010	Unclear	Unclear	Unclear	Low	Low	Low	Low
Liao *et al* (23)	2010	High	Unclear	Unclear	Unclear	Low	Low	Low
Deng *et al* (24)	2011	Unclear	Unclear	Unclear	Low	Low	Low	Low
Peng and Chen (25)	2011	Unclear	Unclear	Unclear	Low	Low	Low	Low
Zhao *et al* (26)	2011	Unclear	Unclear	Unclear	Low	Low	Low	Low
Li and Xu (27)	2012	Unclear	Unclear	Unclear	Unclear	Low	Low	Low

RCTs, randomized controlled trials.

**Table III:** Cosmetic result of included RCTs.

Author (ref.)	Year	Assessment method	Result (C/T)	Conclusion
Kerawala *et al* (11)	2002	VAS	Subjective: 1.5±1.6/2.6±2.1, P=0.13; objective: 2.8±1.3/3.5±1.3, P=0.12	Insignificant difference
Chen and Yang (20)	2004	Doctor observed	Marked/inconspicuous, P<0.01	Significant difference
Asal *et al* (12)	2005	Questionnaire and doctor observed	Subjective: all patients were pleased with the cosmetic result; objective: the facial contours of 7/6 patients were not unsatisfied to the otolaryngologist	Insignificant difference
Zhao *et al* (19)	2005	Questionnaire	22/4 patients felt unsatisfied, P<0.05	Significant difference
Zhi *et al* (21)	2007	Questionnaire	26/4 patients experienced earlobe depression, P<0.05	Significant difference
Ding *et al* (22)	2010	Doctor and patient observed	60/2 patients experienced facial depression, P=0.0014	Significant difference
Liao *et al* (23)	2010	Doctor observed	18/8 patients experienced facial depression, P<0.01	Significant difference
Deng *et al* (24)	2011	Doctor observed and examined	79/6 patients experienced facial depression, P<0.01	Significant difference
Li and Xu (27)	2012	Reported by patient	6/0 patients felt unsatisfied, P<0.05	Significant difference

VAS, 10-cm visual analog scale; RCTs, randomized controlled trials; T, sternocleidomastoid myocutaneous flap groups; C, control groups.
